# Novel insight into Chronic Inflammatory Demyelinating Polineuropathy in APECED syndrome: molecular mechanisms and clinical implications in children

**DOI:** 10.1186/s13052-017-0331-6

**Published:** 2017-01-19

**Authors:** Mariella Valenzise, Tommaso Aversa, Giuseppina Salzano, Giuseppina Zirilli, Filippo De Luca, Maureen Su

**Affiliations:** 1 Department of Human Pathology of Adulthood and Childhood, University of Messina, Via Consolare Valeria-98125, Messina, Italy; 2 Department of Microbiology and Immunology- UNC Hospitals Children’s Specialty Clinic- Chapel Hill, Chapel Hill, NC USA

**Keywords:** AIRE gene, Autoimmune polyendocrinopathy-candidiasis-ectodermal-dystrophy, Molecular pathophysiology, Peripheral neuropathy, Specific therapies

## Abstract

Autoimmune polyendocrinopathy-candidiasis-ectodermal-dystrophy (APECED) is a rare primary immunodeficiency disorder typically caused by homozygous *AIRE* gene mutation. It is characterized by the association of multiple autoimmune diseases, with a classical triad including chronic mucocutaneous candidiasis, hypoparathyroidism and adrenocortical failure. Its clinical spectrum has significantly enlarged in the last years with the apparence of new entities. One of these novel manifestations is the chronic inflammatory demyelinating polineuropathy (CIDP), that is characterized by involvement of peripheral nervous system, with nerve demyelination, progressive muscular weakness of both arms and legs and sensory loss. The identification of myelin protein zero as an important autoantigen (Ag) in CIDP may suggest the development of Ag-based therapies, such as Ag-specific DNA vaccination or infusion of Ag-coupled cells.

## Background

Autoimmune polyendocrinopathy-candidiasis-ectodermal-dystrophy (APECED) is a rare primary immunodeficiency disorder, which is typically caused by homozygous *AIRE* gene mutations [1, 2]. It is a T-cell mediated disease with increased frequencies of CD8+ effectors and reduction of FoxP3 + T regulatory cells. In addition, APECED patients show a significant alteration of the B-cell phenotype and a dysregulation of the B-cell function involving peripheral innate immune mechanisms, particularly those with longer disease duration [3].

APECED is characterized by the association of multiple autoimmune diseases, with a classical triad including chronic mucocutaneous candidiasis, hypoparathyroidism and adrenocortical failure. Its clinical spectrum has significantly enlarged in the last years [4]. In fact, besides the classical triad, many other endocrine and non-endocrine autoimmune manifestations, several of which are associated with significant morbidity or mortality, may occur in this condition and vary significantly, even inside the same families and in children with the same *AIRE* mutations [5–7]. One of these novel diseases, i.e., the chronic inflammatory demyelinating polineuropathy (CIDP), is characterized by an involvement of peripheral nervous system with nerve demyelination, progressive muscular weakness of both arms and legs and sensory loss [7].

The aims of this review are: 1) to highlight the molecular aspects of CIDP in mice and humans; 2) to report the most recent views on its pathogenesis and 3) to alert pediatricians to the unusual neurological signs of the disease that may suggest CIDP diagnosis in children.

### Mechanisms of Aire action

**Fig. 1:**
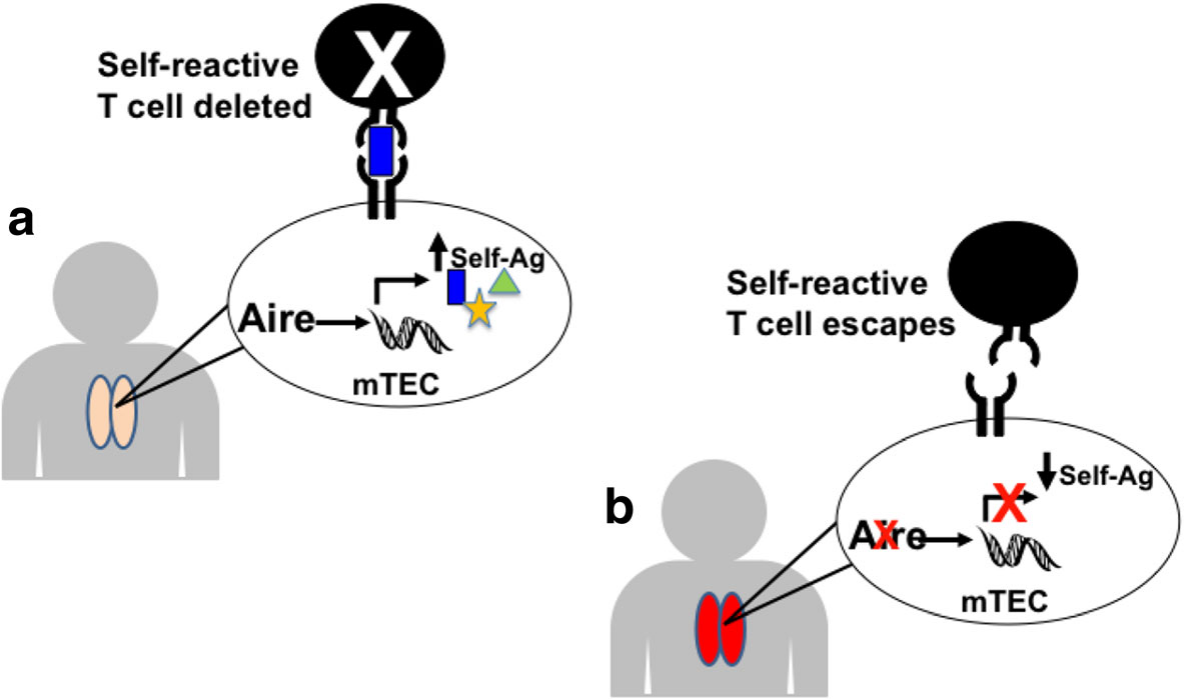
**a** and **b** Aire promotes expression of self-antigens in mTECs and deletion of self-reactive T cells within the thymus. **a** In a normal thymus, Autoimmune Regulator (Aire) upregulates expression of a large number of tissue-specific self-antigens (Self-Ag) within medullary thymic epithelial cells (mTECs). Developing T cells which recognize these self-antigens with high affinity are eliminated to prevent these T cells from causing autoimmunity. **b** In Aire deficient individuals, lack of Aire expression within mTECs results in decreased expression of tissue-specific self-antigens in mTECs. Lack of presentation of these self-antigens to self-reactive T cells allows escape of these self-reactive T cells from clonal deletion. Release of self-reactive T cells into the periphery promotes autoimmunity

Aire promotes T cell tolerance to self-antigens by upregulating the ectopic expression of a wide array of tissue-specific self-antigens in medullary thymic epithelial cells (mTECs) within the thymus [8]. Upregulation of self-antigens in mTECs promotes the deletion of developing T cells that recognize these self-antigens with high affinity (Fig. 1a). In patients and mice with mutations in *AIRE*, loss of Aire function results in decreased expression of tissue-specific self-antigens by mTECs and defective negative selection of self-reactive T cells (Fig. 1b) [9]. Escape of these self-reactive T cells into the periphery predisposes to the development of autoimmunity against multiple organs.

In addition to its role in the negative selection of effector T cells, Aire has been implicated also in other functions, which may promote self-tolerance. For example, Aire has been postilated to play a role in antigen presentation within the thymus [9] and to affect the frequency and function of regulatory T cells (Tregs), which are T cell subsets with immunosuppressive function. Thus, Aire may prevent autoimmunity by affecting a number of immunoregulatory mechanisms.

Since the translated Aire protein is involved in the transcriptional regulation of the expression of organ-specific antigens within the thymus, the fundamental pathogenetic mechanism, in APECED patients, appears to be T cell mediated [10].

### NOD mice heterozygous for the Aire G228W mutation develop spontaneous autoimmune peripheral polyneuropathy

A link between Aire deficiency and the development of autoimmune peripheral neuropathy was made in mice harboring the *AIRE* G228W mutation on the autoimmune-prone non-obese diabetic (NOD) background. The *AIRE* G228W point mutation was originally described in an Italian kindred with Mendelian inheritance of autoimmunity and differs from most *AIRE* mutations in its dominant inheritance pattern and distinct pattern of autoimmunity. To study the effects of this mutation on Aire function, we engineered knockin mice with the G228W mutation targeted by homologous recombination to the *AIRE* locus [11]. Similar to patients with one copy of this mutation, heterozygous mice (*Aire* ^*GW/+*^ mice) develop spontaneous autoimmunity in a pattern distinct from *Aire* ^*o/o*^ mice. *Aire* ^*GW/+*^ mice have partial loss of Aire function in that mTECs retain approximately 10% of normal levels of tissue-specific self-antigen expression . This residual antigen expression appears to be sufficient to protect from certain autoimmune diseases that develop in *Aire* ^*o/o*^ mice, including those associated with early lethality on the NOD background. Survival after the first months may allow development of autoimmune manifestations with later onset, including spontaneous autoimmune peripheral polyneuropathy (SAPP).

Of note, the dominant *AIRE* G305S mutation has also been associated with the development of neuropathy in a patient cohort [12]. It is important to underline, however, that patients with two copies of *AIRE* mutations have also been noted to develop neuropathy. Thus, the development of autoimmune peripheral neuropathy does not appear to be specific to dominant *AIRE* mutations but can occur in patients who have either one dominant *AIRE* mutation or two copies of *AIRE* mutations.

### SAPP in *NOD.Aire*^*GW/+*^ mice shares multiple features with CIDP in humans

By 20 weeks of age, approximately 80% of female *Aire* ^*GW/+*^ mice on the NOD background (*NOD.Aire* ^*GW/+*^ mice) develop spontaneous neuropathy [11, 13] that is not seen in wildtype (*NOD.WT*) littermates. Affected mice display bilateral weakness of the hind limbs that may subsequently progresses to severe paralysis of all limbs. This neuropathy is associated with immune cell infiltration in *NOD.Aire* ^*GW/+*^ sciatic nerves, but not in brain or spinal cord.

In order to demonstrate the importance of immune cells in the pathogenesis of this neuropathy, we transferred spleen and lymph node cells from neuropathic *NOD.Aire* ^*GW/+*^ mice into immunodeficient NOD *Prkdc* ^*scid/scid*^ (*NOD.scid*) mice. All recipients developed neuropathy by 7 weeks, suggesting that *NOD.Aire* ^*GW/+*^ immune cells are pathogenic [13]. Furthermore, recipients of purified CD4+ T cells also developed neuropathy that was accompanied by immune cell infiltration of sciatic nerves [12], suggesting that CD4+ T cells are sufficient to transfer SAPP.

SAPP in *NOD.Aire* ^*GW/+*^ mice shares a number of features with CIDP. First, CD4+ T cells and F4/80+ macrophages are abundant immune cell types in CIDP patient nerve biopsies. Similarly, CD4+ T cells and F4/80+ macrophages are also prevalent in the immune infiltrates of *NOD.Aire* ^*GW/+*^ sciatic nerves [13]. Second, CD4+ helper T cells in CIDP patients are skewed toward the production of interferon (IFN) gamma. Similarly, approximately 40% of CD4+ helper T cells in the sciatic nerve infiltrates of neuropathic *NOD.Aire* ^*GW/+*^ produce IFN gamma after in vitro stimulation and less than 1% produce IL-17 or IL-4 [12]. Third, increased expression of the chemokine CXCL10 (IP-10) in sural nerve biopsies is associated with CIDP. In neuropathic *NOD.Aire* ^*GW/+*^ mice, CXCL10 expression is also increased in the sciatic nerves. Finally, demyelination is a hallmark of CIDP, and extensive demyelination is characteristic of sciatic nerves from neuropathic *NOD.Aire* ^*GW/+*^ mice [13].

Thus, SAPP in this mouse model recapitulates a number of features of human CIDP.

### Defective central tolerance to P0 underlies the immune tolerance defect in *NOD.Aire*^*GW/+*^ mice

A key question in autoimmune disease is the identity of inciting antigens. This question has been particularly difficult to address in CIDP because only a minority of patient sera samples contain PNS antigen-specific autoantibodies. Aire deficiency, on the other hand, has been associated with autoantibody production against multiple organs in both mice and humans [7]. We therefore utilized *NOD.Aire* ^*GW/+*^ mice as a strategy to identify potential relevant peripheral nerve antigens important in SAPP [13]. Using autoantibodies present in the sera of neuropathic *NOD.Aire* ^*GW/+*^ mice, we isolated an antigen that appeared to be recognized by all PNS-reactive serum specimens from *NOD.Aire* ^*GW/+*^ mice. This antigen was identified by mass spectrometry to be Myelin Protein zero (P0), a 28 kD protein which compromises the majority of peripheral nerve myelin.

P0 is a known autoantigen in PNS autoimmunity in various other settings. Although autoantibodies against PNS antigens occurs in only a minority of CIDP patients, the most frequent autoantibody specificity is against P0. In mice other animals, immunization with P0 is sufficient to provoke Experimental Autoimmune Neuropathy (EAN), an induced model of autoimmune peripheral neuropathy that has been widely used to study mechanisms of disease. Finally, P0 has been identified as an important target antigen in spontaneous PNS autoimmunity which develops in NOD mice deficient for the costimulatory molecule B7-2, and NOD mice engineered to express T cells with specificity against P0 develop early onset, fulminant neuropathy.

Based on our working model of Aire action (Fig. 1), we hypothesized that P0 is also expressed within mTECs and therefore promotes clonal deletion of P0 specific T cells developing within the thymus. Furthermore, we hypothesized that thymus expression of P0 would be under the control of Aire, so that decreased P0 expression would be detected in mTECs from Aire deficient mice. In support of this, P0 expression was indeed detectable within mTECs and detected at approximately 10% of wildtype levels in mTECs derived from *NOD.Aire* ^*GW/+*^ mice [12]. Additionally, as would be expected if lack of clonal deletion was occurring in Aire-deficient thymus, increased T cell reactivity to P0 was seen in the spleen of neuropathic *NOD.Aire* ^*GW/+*^ mice compared with wildtype counterparts [12]. Thus, lack of Aire-mediated expression of P0 in mTECs is associated with increased immune response to P0 and development of spontaneous autoimmunity against the PNS.

### P0 is also an autoantigen in APECED patients with CIDP

In people, autoimmune peripheral neuropathy manifests most commonly as CIDP or Guillan Barre Syndrome. These diseases occur with a combined prevalence of between 2 and 8 per 100,000. The pathophysiology of autoimmune peripheral neuropathy was not well understood, and, importantly, the pathogenic specific antigen of autoimmune peripheral neuropathy was not known. In fact, if there is general agreement that CIDP is an autoimmune disorder, significant questions remain concerning disease mechanisms and target antigens.

CIDP typically presents as a progressive or relapsing, symmetrical mainly motor neuropathy with proximal and distal weakness. The diagnosis of CIDP usually depends upon the electrophysiological studies showing features consistent with demielination as prolongation of distal latency, or reduction in motor conduction velocity, or prolongation of F wave latency, or absence of F waves, or partial conduction block, or abnormal temporal dispersion, or distal CMAP duration and one other demyelinating parameter in one other nerve. Supportive features for a diagnosis of CIDP include: elevated cerebrospinal fluid proteins without an increased leukocyte count; nerve biopsy finding of primary demyelination or improvement following immunotherapy.

As mentioned previously, two unrelated patients with mutations in *AIRE* also spontaneously developed CIDP.

The boys described by our group [7] showed progressive muscular weakness involving both arms and legs, which was associated with sensory loss and absent tendon reflexes.

Electromyograhy (EMG) revealed neurogenic changes with denervation in the intrinsic muscles of the hands. Motor and sensory nerve conduction velocities were reduced in both ulnar nerves and in the right median nerve, with signs of chronodispersion.

Sural nerve biopsy, performed in one of these patients showed moderate demyelination in all nerve fascicles, patchy CD4-, CD8- and CD68-positive cell infiltration around two small endoneural blood vessel walls and moderate subperineural oedema.

No serum autoantibodies against either myelin-associated glycoprotein or peripheral nerve myelin proteins (GM1 gangliosides and onco-neural antigens-HU), CV2, amphyphysin) [6]. By radioligand binding assay, however, both of these patients demonstrated autoantibodies against P0, suggesting that P0 is an important antigen in both mice and humans deficient in Aire [13].

Current treatments consist primarily of glucocorticoids, intravenous immunoglobulin (I.V. Ig) and plasmapheresis, which result in non-specific immunosuppression. Recently, subcutaneous immunoglobulins (SC-Ig) has received increasing attention because its efficacy seem to be similar to I.V.Ig [14–16]. In those patients, neurological symptoms and electrophysiological tests significantly improved after an initial therapy with I.V.Ig (400 mg/kg per day for 5 days; therapy regimen was repeated every 3 weeks for two months) followed by one with oral prednisone. After relapse the patient two has started treatment with SCIg with an increase in autonomy and quality of life through self treatment. Despite this advance with SCIg, more specific therapies that target PNS autoimmunity remains an unmet clinical need.

## Conclusions

1. MP0 is a target of autoimmunity in APECED children;
2. therefore, AIRE-mediated autoimmune peripheral neuropathy should be included among the components of this syndrome;
3. the identification of P0 as an important autoantigen in CIDP may suggest the development of Ag-based therapies, such as Ag-specific DNA vaccination or infusion of Ag-coupled cells;
4. the demonstration of the altered B cell phenotype in APECED patients may suggest the possible utility of future anti B-cell treatments such as Rituximab in order to control an underlying immunological defect therefore avoid the disease progression to multiorgan autoimmunity.

## Abbreviations

AIRE: AutoImmune REgulator
APECED: Autoimmune polyendocrinopathy-candidiasis-ectodermal-dystrophy
CIDP: Chronic inflammatory demyelinating polineuropathy
CMAP: Compound muscle action potentials
EAN: Experimental autoimmune neuropathy
EMG: Electromyograhy
GBS: Guillan Barre Syndrome
IVIG: Intravenous immunoglobulins
MP0: Myelin protein zero
mTECs: Medullary thymic epithelial cells
NOD: Non-obese diabetic
PNS: Peripheral nervous system
SAPP: Spontaneous Autoimmune Peripheral Polyneuropathy
SCIg: Subcutaneous immunoglobulin
Tregs: Regulatory T cells
